# Characteristics of duplicated gene expression and DNA methylation regulation in different tissues of allopolyploid *Brassica napus*

**DOI:** 10.1186/s12870-024-05245-8

**Published:** 2024-06-08

**Authors:** Weiqi Sun, Mengdi Li, Jianbo Wang

**Affiliations:** 1 State Key Laboratory of Hybrid Rice, College of Life Sciences, Wuhan University, Wuhan, 430072 China; 2 Key Laboratory of Resource Biology and Biotechnology in Western China, Ministry of Education, College of Life Sciences, Northwest University, Xi’an, 710069 China

**Keywords:** *Brassica napus*, DNA methylation, Gene expression, Four tissues, Duplicated genes

## Abstract

Plant polyploidization increases the complexity of epigenomes and transcriptional regulation, resulting in genome evolution and enhanced adaptability. However, few studies have been conducted on the relationship between gene expression and epigenetic modification in different plant tissues after allopolyploidization. In this study, we studied gene expression and DNA methylation modification patterns in four tissues (stems, leaves, flowers and siliques) of *Brassica napus*and its diploid progenitors. On this basis, the alternative splicing patterns and *cis*-*trans* regulation patterns of four tissues in *B. napus* and its diploid progenitors were also analyzed. It can be seen that the number of alternative splicing occurs in the *B. napus* is higher than that in the diploid progenitors, and the IR type increases the most during allopolyploidy. In addition, we studied the fate changes of duplicated genes after allopolyploidization in *B. napus*. We found that the fate of most duplicated genes is conserved, but the number of neofunctionalization and specialization is also large. The genetic fate of *B. napus* was classified according to five replication types (WGD, PD, DSD, TD, TRD). This study also analyzed generational transmission analysis of expression and DNA methylation patterns. Our study provides a reference for the fate differentiation of duplicated genes during allopolyploidization.

**Supplementary Information:**

The online version contains supplementary material available at 10.1186/s12870-024-05245-8.

## Introduction

Whole-genome duplication (WGD) events that directly double chromosomes, are thought to be a driving force for species differentiation. Studies have found that polyploidy is very frequent in the evolution of flowering plants, and doubling events have occurred in both existing angiosperms and seed plants before differentiation, which may have contributed significantly to the production of flowers and seeds [1]. For example, studies have confirmed that *Arabidopsis* has undergone at least three rounds of WGD [2] and poplar (*Populus trichocarpa*) at least two rounds of WGD [3]. In addition to these two ancient WGD events, maize experienced its most recent round of WGD about 5 million to 12 million years ago [4]. Most newly formed polyploids undergo a differentiation process that includes gene losses, genome rearrangement, and epigenetic changes [5]. Theoretical studies maintain that the most likely fate of duplicated genes is the loss or pseudogenization of one of them [2]. However, several genome-wide analyses have shown that many duplicate copies may survive long after WGD, and over retention of duplicated copies of WGD in plant genomes across gene families has been shown to be non-random [6]. The genes retained in duplicates are not evenly distributed in different functional categories. Some genes are able to duplicate, while others revert to a single gene state. For instance, genes encoding transcription factors, protein kinases, and ribosomal proteins were found to be over retained after the most recent round of WGD in Arabidopsis [7]. The pattern of functional bias in duplicated genes can be detected in other lineages as well, such as poplar, rice, and maize [8]. In concert, these observations suggest some underlying mechanisms may have played a role in determining the evolutionary fates of duplicated genes resulting from WGD events. The modes of gene replication include small-scale replication (SSD) and whole genome duplication (WGD) [9], among which SSD includes tandem duplication (TD), transposed duplication (TRD), dispersed duplication (DSD), proximal duplication (PD).

Firstly, either benefiting from increased gene dosage [10] or recombination between gene copies [11] can result in conservation, whereby both copies retain their ancestral function. Second, the acquisition of new functions by one gene copy may lead to neofunctionalization, which occurs when beneficial mutations occur in one copy, while maintaining the ancestral function in the other copy [10]. Third, deleterious mutations targeting different functional domains of each gene copy can result in subfunctionalization, in which each copy retains a subset of its ancestral function [12]. Fourth, a combination of deleterious and beneficial mutations may lead to specialization, whereby each copy retains a subset of the ancestral function while acquiring a new one [13, 14]. Despite the fact that mutations initiating the latter three retention mechanisms may take a while to manifest, dosage balance can serve as an intermediate state for preventing gene loss due to nonfunctionalization during this waiting period [15–19]. Polyploidization increases epigenome complexity and transcriptional regulation, resulting in genome evolution and greater adaptability to varying environments [20].

More than 70% of angiosperms have polyploidized over their evolutionary history, and polyploids have both phenotypic and physiological advantages over diploid progenitors [21, 22]. Natural polyploids are formed in two ways, autopolyploidization and allopolyploidization [23]. Polyploidy often brings a certain impact on the plant genome, especially allopolyploidy. The parental chromosome sets are non-homologous, so in addition to genome doubling, genome hybridization also affects the plant genome, leading to changes over time [24, 25]. In addition to genomic changes, the newly formed polyploids also showed a series of changes in other aspects, including transcriptome changes and epigenetic changes. During and after allopolyploidization, genetic and epigenetic changes occur extensively [26]. As a result of all of these changes, nascent allopolyploids may be established more successfully, ecological diversity may increase, adaptation to and expansion into new geographic niches may occur, and community structure may also be altered [7, 27–29].

As the largest allotetraploid oil crop in the world, *Brassica napus* also serves as a model plant for studying polyploid morphology, evolution, and genomics. About 7500 years ago, it was formed by interspecific hybridization between species with the A-genome, *Brassica rapa* (2n = 20, A_r_A_r_), and a C-genome species, *Brassica oleracea* (2n = 18, C_o_C_o_) [30]. The release of the *B. napus* reference genome in recent years has accelerated research on *B. napus* functional genomics and breeding programs [31–33]. As allotetraploid, *B. napus* is an excellent material for studying polyploid problem. During the process of polyploidy, a large number of gene replication events occur. But what the fate of these duplicating genes is, and how they are regulated by epigenetics, is what we are concerned about. Some studies have indicated that the content of GC3 (GC content of the third codon), a repeating gene of coevolution, has increased the GC3 (GC content of the third codon) content of duplicated genes that underwent concerted evolution was elevated [34]. In plants, genes with high GC3 content may provide more targets for DNA methylation, resulting in a high degree of gene expression variability [35]. As a result, it is likely that duplicated genes with high GC3 content also possess more targets for DNA methylation [36]. At present, there are few studies on the regulation of epigenetic modification on duplicate genes after allopolyploidization. We analyzed gene expression, alternative splicing, *cis-trans* regulation and DNA methylation modification patterns in four tissues (stems, leaves, flowers and siliques) of *B. napus* and its diploid progenitors. Further, this study also analyzed generational transmission analysis of expression and DNA methylation patterns. In addition, we studied the fate changes of duplicated genes after allopolyploidization in *B. napus*. In this study, we performed transcriptomic and global genomic methylation analysis of *B. napus* and its two diploid progenitors, to explore the possible molecular basis for the successful establishment and adaptation of allopolyploid *B. napus*. To provid a reference for epigenetic regulation and fate differentiation of duplicated genes during allopolyploidization.

## Materials and methods

### Material planting and collection

Three plant materials, including the natural allotetraploid *B. napus* (cv. Darmor, 2n = 4x = 38, A_n_A_n_C_n_C_n_) and its two diploid progenitors, *B. rapa* (cv. Chiifu, 2n = 20, A_r_A_r_) and *B. oleracea* (cv. Jinzaosheng, 2n = 18, C_o_C_o_), were obtained from the Oil Crops Research Institute, Chinese Academy of Agricultural Sciences, China. The plants were randomly planted under natural conditions (outdoors) in the greenhouse at Wuhan University, China. Soil is collected locally, mixed with compound fertilizer, and watered manually to keep the soil moist. Pollinate each plant material by hand. To prevent contamination by foreign pollen, parts of the inflorescence are bagged before flowering. At the same time, the inflorescence stems, young leaves, blooming flowers and siliques (10 DAP, days after pollination) of 6-month-old plants were collected (10:00 am), and were rapidly frozen in liquid nitrogen to extract RNA. Three biological replicates were performed.

### Sequencing and data analysis

According to the manufacturer’s protocol, total RNA was extracted using TRIzol reagent (Invitrogen, USA) according to the manufacturer’s protocol and then treated with RNase-free DNase I (Thermo Scientific, USA) to remove the residual DNA. The production and purity of total RNA were assessed using the Agilent 2100 Bioanalyzer (Agilent RNA 6000 Nano Kit). RNA extraction criteria for RNA-seq library building, including the content of total RNAs ≥ 1 µg, the concentration of total RNAs. Using cetyl trimethylammonium bromide (CTAB), genomic v DNA was isolated from 12 samples. A Zymo Research, Irvine, CA, USA kit was used for bisulfite treatment. Acegen Bisulfite-Seq Library Prep Kit (AG0311; Acegen, Shenzhen, China) was used to prepare WGBS libraries, and Illumina HiseqX10 (30-fold sequencing depth) was used to sequence them. By mixing WGBS reads of *B. rapa* and *B. olerocea*, an in-silico hybrid was constructed, and the data size of the hybrid was consistent with *B. napus’s*. As part of the mapping process, raw reads were filtered using TRIMMOMATIC (v.0.36), and clean reads were mapped to the reference genome (Brana_Dar_V5, http://www.brassicadb.cn/#/Download/). using BSMAP (v.2.73).

MACSE was used to align duplicates with ancestral single-copy genes, which incorporates frameshifts and stop codons. Based on a codeml package in PAML 4.0 with run mode = 10, model = 0, and NS sites = 0, we computed Ka, Ks, and Ka/Ks. For reasons of avoiding saturation, we only took into consideration genes with Ks < 3. Gene expression abundances in four tissues (stem, leaf, flower, siliques) estimated from RNA-seq data of *B. rapa*, *B. oleracea*, and *B. napus* were downloaded from Expression Atlas at https://www.ebi.ac.uk/gxa/home. Based on RNA-seq data, HTSeq 0.6 quantifies reads containing unambiguous mappings to a single gene, minimizing the chance of incorrect mappings. Data were then log-transformed, and genes with log_2_(FPKM + 1) < 1 in all four tissues were removed, as such genes are expressed at low levels that may be attributed to transcriptional noise. We estimated the expression breadth of each gene with the tissue specificity index τ, which is defined as where xi represents the expression level in the i tissues normalized by the maximal expression value. Tissue specificity increases as τ, ranging from 0 to 1, becomes larger.

The CDROM R package, which implements Assis and Bachtrog’s phylogenetic method, was used to classify retention mechanisms for duplicate genes in our dataset. The CDROM takes as input tables of expression measurements for multiple conditions in two sister species, lists of orthologous single-copy genes in the two sisters, and a list of parent (P) and child (C) duplicate gene pairs in one sister and their ancestral (A) genes in the second sister. To analyze the RNA-seq data described above, which consists of log-transformed TPMs for genes in four tissues of *B. rapa*, *B. oleracea*, and *B. napus*, we used *B. rapa* as the sister species to *B. oleracea* and *B. napus*. As part of its first step, the CDROM calculates Euclidian distances between the expression profiles of orthologous single copies, the expression profiles of parent and child duplicate genes, and the ancestral gene (EP, A and EC, A) and combined expression profiles of both duplicate genes and the ancestral gene (E_P + C, A_). The next step is to classify duplicate retention mechanisms using a user-specific cutoff for E_S1, S2_ (E_div_). A functionally conserved duplicate contains E_P, A_ + E_div_ and E_C, A_ + Ediv; those with either E_P, A_ ≤ E_div_ and E_C, A_ > Ediv or E_C, A_ ≤ E_div_ and E_P, A_ > E_div_ as neofunctionalized; those with E_P, A_ > E_div_, E_C, A_ > E_div_, and E_P + C, A_ ≤ Ediv as subfunctionalized, and those with E_P, A_ > E_div_, E_C, A_ > E_div_ and E_P + C, A_ > E_div_ as specialized. To select Ediv for each species, we used Euclidian distance distributions between gene expression profiles. CDROM first calculates Euclidian distances between expression profiles of orthologous single-copy genes (E_S1, S2_), expression profiles of parent and child duplicate genes and the ancestral gene (EP, A and E_C, A_), and combined expression profiles of both duplicate genes and the ancestral gene (E_P + C, A_). Next, it uses a user-specific cutoff for ES1, S2 (E_div_) to classify retention mechanisms of duplicates. Specifically, duplicates with E_P, A_ ≤ E_div_ and E_C, A_ ≤ E_div_ are classified as functionally conserved; those with either E_P, A_ ≤ E_div_ and E_C, A_ > E_div_ or E_C, A_ ≤ E_div_ and E_P, A_ > Ediv as neofunctionalized; those with E_P, A_ > E_div_, E_C, A_ > E_div_, and E_P + C, A_ ≤ E_div_ as subfunctionalized, and those with E_P, A_ > E_div_, E_C, A_ > E_div_ and E_P + C, A_ > E_div_ as specialized. We used distributions of Euclidian distances between gene expression profiles to choose E_div_ for each species.

### Analysis of differentially expressed genes (DEGs) and gene annotation

For this study, the DESeq2 (version: DESeq2_1.20.0) method was used to identify DEGs, and genes with |log_2_ fold change| ≥ 1 and padj (adjusted P value) ≤ 0.001 were defined as DEGs in this study. Annotations of all genes were performed using eggNOG-mapper using eggNOG 4.5 orthology data [37, 38]. An online functional classification of genes based on the Gene Ontology (GO) was performed using the website WEGO 2.0 (http://wego.genomics.org.cn) [39].

### Characterization of *cis/trans* regulation effects

According to previous studies, *Cis* and *trans* effects were distinguished using the read counts of in silico ‘hybrid’ (A_r_-C_o_), resynthesized and *B. napus* from RNA sequencing a were used to distinguish *Cis* and *trans* effects [40–42]. These two effects co-regulated read count divergence among homoeologous gene pairs in progenitors/parents (represented by A_r_-C_o_); thus, these two effects together were measured by the log_2_ ratios of the read count divergences, such that A = log_2_(A/C). Due to their common *trans* environment, *cis* effects were measured by reading count divergences of homoeologous gene pairs in progenies (A-C/AACC; B = log_2_(A_n_/C_n_)). *Trans* effects were determined by subtracting the expression divergences of gene pairs in progenies from those of progenitors/parents (A-B). The gene pairs were divided into seven regulatory categories according to the statistical results of A vs. C (A = l ≠ 0), A_n_ vs. C_n_ (B = l ≠ 0), and A vs. B (A = l ≠ B). Significant expression divergences of gene pairs in progenitors/p6arents (A ≠ 0) and *cis* effects (B ≠ 0) were determined using DESEQ2 [32], as discussed in the sub-section ‘Biased expression analysis of gene pairs’, above, and the trans effects (A ≠ B) were tested using Student’s t-test, followed by the adjustment of P-values using FDR (FDR < 0.05). GGPLOT2 was used to create the line graph and boxplot.

### The statistical tests

This study tested the statistical significance of each comparison using R (R Foundation for Statistical Computing, Vienna, Austria; https://www.r-project.org), and several statistical tests were used, including the exact binominal test, Chi-squared test, Kolmogorov–Smirnov test (K-S test), Student’s t-test, Wilcoxon rank sum test, and Pearson’ s product-moment correlation.

## Results

### Retention mechanisms of WGD- and SSD- derived duplicates in *B. napus* homoeologs

In order to classify the retention mechanism of WGD and SSD- derived *Brassica* duplicated genes, we applied the phylogenetic method developed by Assis and Bachtrog to expression profiles constructed from RNA-seq data in four tissues of single-copy, ancestral, parent, and child genes of *B. rapa*, *B. oleracea*, and *B. napus*. In particular, the method first uses the Euclidian distance distribution between single-copy gene expression profiles to establish a cutoff point representing expected expression differences between two species. Next, it calculates the Euclidian distance between ancestor and parent expression profiles, ancestor and child expression profiles, and ancestor and combined parent-child expression profiles. Finally, the retention mechanisms of each pair of repeats were classified according to phylogenetic rules. In short, ancestral gene expression profiles should be similar to those of the parent and child under conservation, to those of one copy but not the other under neofunctionalization, and to those of neither copy under subfunctionalization or specialization.

**Fig. 1:**
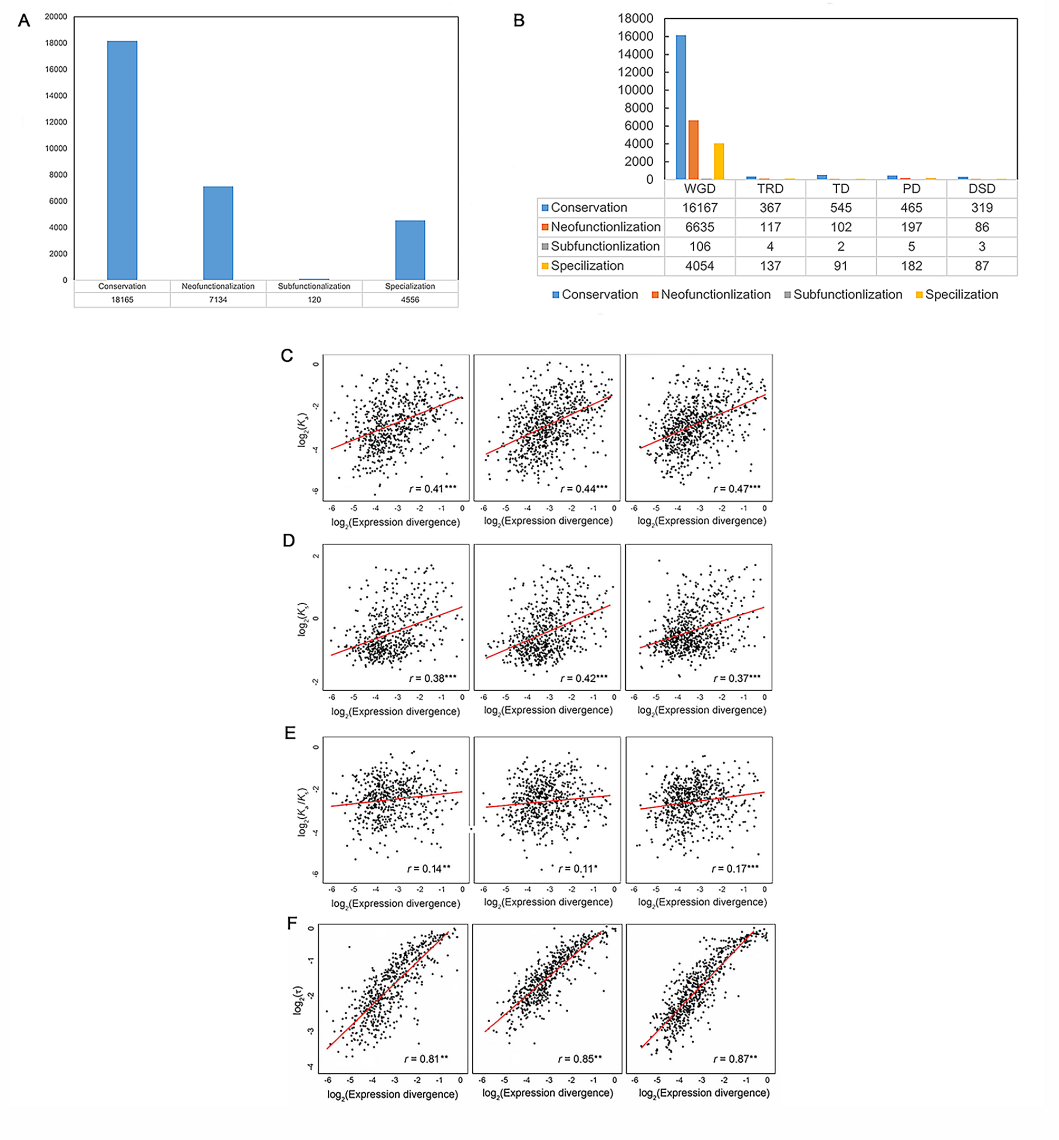
(**A**)The four fates of duplication genes in *B. napus.* (**B**) Classified the genetic fates of *Brassica napus*and its diploid progenitors according to five types of replication (WGD, PD, DSD, TD, TRD). Relationship between expression and protein-coding sequence divergence rates of *Brassica* duplicate genes. Scatterplots showing correlations between expression divergence (Euclidian distance) and (**C**) nonsynonymous sequence divergence (Ka), (**D**) synonymous sequence divergence (Ks), and (**E**) nonsynonymous/synonymous sequence divergence (Ka/Ks) rates of SSD-derived duplicate genes in *B. rapa* (left) and *B. napus* (right), *B. oleracea* (middle). The best-fit linear regression line is shown in red, and Pearson’s correlation coefficient (*r*) is provided at the bottom right, for each panel. *P < 0.05, **P < 0.01, ***P < 0.001. (**F**) Relationship between expression divergence and tissue specificity of *Brassica* duplicate genes. a Scatterplot showing correlation between expression divergence (Euclidian distance) and tissue specificity (τ) of *Brassica* duplicate genes in *B. rapa* (left) and *B. napus* (right, *B. oleracea* (middle). The best-fit linear regression line is shown in red, and Pearson’s correlation coefficient (*r*) is provided at the bottom right, for each panel

A comparison of ancestral and combined parent-child expression profiles is necessary to distinguish between subfunctionalization and specialization. In the case of similar expression profiles, they indicate subfunctionalization of the ancestral gene between parent and child copies, while differences indicate functional differences between the three genes due to specialization. Application of the described classification method revealed similar proportions of each retention mechanism in WGD and SSD- derived duplicates of *B. rapa*, *B. oleracea*, and *B. napus.* Thus, after SSD, the genes of the three *Brassica* species appear to have undergone a similar evolutionary path. Overall, about 60% of WGD- and SSD- derived *Brassica* duplicates are conserved, 24% are neofunctionalized, 0.5% are subfunctionalized, and 15.5% are specialized (Fig. 1A). Therefore, conservation is the most common retention mechanism, suggesting that SSD generally leads to increased gene dosage in *B. napus* and its diploid progenitors.

Then the genetic fate of *B. napus* and its diploid progenitors were classified according to 5 replication types (WGD, PD, DSD, TD, TRD; Fig. 1B). Previous studies have shown that expression divergence is often positively correlated with protein-coding sequence divergence and tissue specificity of duplicate genes increased tissue specificity [43]. To assess this relationship in *Brassica*, we calculated Pearson’s correlation coefficients (r) between expression divergence (Euclidian distance) and nonsynonymous sequence divergence (Ka), synonymous sequence divergence (Ks), nonsynonymous-to-synonymous sequence divergence (Ka/Ks) rates, and tissue specificity index (τ) of each SSD-derived duplicate gene and its ancestral gene in *B. rapa*, *B. oleracea*, and *B. napus*. In three species, the divergence of expression was moderately positive correlated with Ka/Ks (Fig. 1C; *r* = 0.41 − 0.47; *P* < 0.001), weakly positive correlated with Ka/Ks (Fig. 1D; *r* = 0.37 − 0.42; *P* < 0.001), and weakly positive correlated with Ks (Fig. 1E; *r* = 0.11 − 0.17; *P* < 0.05), strongly positive correlated with tissue specificity index (τ) (Fig. 1F; *r* = 0.81 − 0.87; *P* < 0.001). Therefore, the expression divergence of SSD-derived duplicates is correlated with protein-coding sequence and tissue specificity, indicating that the expression patterns of Brassica duplicate genes and the coding protein evolve in tandem, and increased expression divergence of SSD-derived Brassica duplicates is associated with greater tissue specificity.

### Differentially expressed genes (DEGs) between *B. napus* and its diploid progenitors in four tissues

**Fig. 2:**
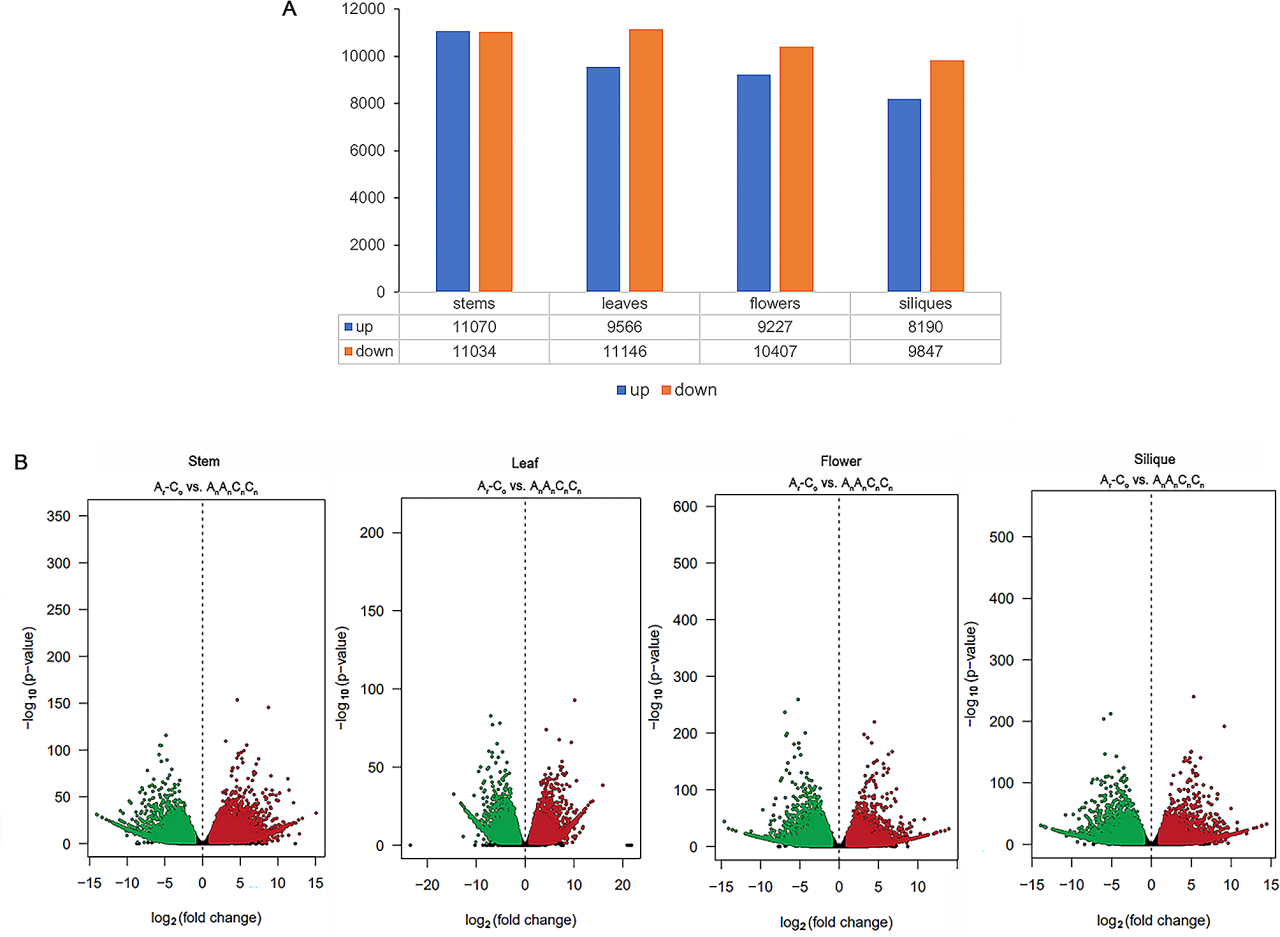
(**A**) The differences in gene expression between allotetraploid *B. napus* (A_n_A_n_C_n_C_n_) and its diploid progenitors (represented by Ar-Co). Blue is down, orange is up, all DEGs in stems, leaves, flowers, and siliques were identified using DESeq2, with |log_2_ fold change| ≥ 1 and padj ≤ 0.001. (**B**) Distribution map of differential genes between allotetraploid *B. napus* (A_n_A_n_C_n_C_n_) and its diploid progenitors (represented by A_r_-C_o_). The distribution of differentially expressed genes was demonstrated by the expression distribution map (combined with *P* value and multiples) and volcano map. Differential gene screening conditions were as follows: difference multiples greater than or equal to 2 and q-value (or FDR) less than or equal to 0.01. Each dot represents a gene, and the green and red dots represent significantly differentially expressed genes. The red dots indicate that the gene expression is up-regulated, the green dots indicate that the gene expression is down-regulated (the treated sample is compared to the control sample), and the gray dots indicate that these genes are not significantly different. The horizontal coordinate represents the multiple of difference (treatment/contrast, logarithm), and the vertical coordinate represents -log_2_ (q-value)

To investigate the gene expression differences between allotetraploid *Brassica napus* (A_n_A_n_C_n_C_n_) and its diploid ancestors (ArAr and CoCo), we identified all DEGs in stems, leaves, flowers and fruits by DESeq2. To investigate the gene expression differences between *B. napus* (A_n_A_n_C_n_C_n_) and its diploid progenitors (A_r_A_r_ and C_o_C_o_), we identified all DEGs in stems, leaves, flowers and siliques by DESeq2. The screening criterion are |log_2_ fold change| ≥ 1 and padj ≤ 0.001. Compared with diploid progenitors (A_r_-C_o_), a total of 81,064 DEGs were identified in four tissues, including 11,070 (up) and 11,034 (down) in stems, 9566 (up) and 1146 (down) in leaves, 9277 (up) and 10,407 (down) in flowers, 8190 (up) and 9847 (down) in siliques (Fig. 2A), the most different tissue was stems. Using the eggNOG database, all genes from the A and C genomes were functionally annotated in four selected tissues to further investigate gene functional differences. The distribution of differentially expressed genes was demonstrated by the expression distribution map (combined with *P* value and multiples) and volcano map (Fig. 2B). Each dot represents a gene, and the green and red dots represent significantly differentially expressed genes. A total of 14.63% (3233 out of 22,104) of genes in stems, 15.41% (3192 out of 20,712) of genes in leaves, 15.63% (3076 out of 19,634) of genes in flowes and 16.51% (2978 out of 18,037) of genes in siliques were labeled to at least one GO item. GO functional classification analysis (WEGO) was performed on all DEGs between *B. napus* and its diploid progenitors. The enrichment of the 25 GO terms with the highest -log_10_ (significance test) value in the four tissues was selected and shown in Fig. S1. In stems and leaves, ATP binding (GO:0040007) is significant enrichment GO term. In flowers, there were three significant enrichment GO terms, including heme binding (GO:0020037), oxidoreductase activity (GO:0016491), and iron ion binding (GO:0005506). In siliques, there were two significant enrichment GO terms, including oxidoreductase activity (GO:0016491), and iron ion binding (GO:0005506) (Fig. S1).

### Alternative splicing features in *B. napus* and its diploid progenitors of four tissues

**Fig. 3:**
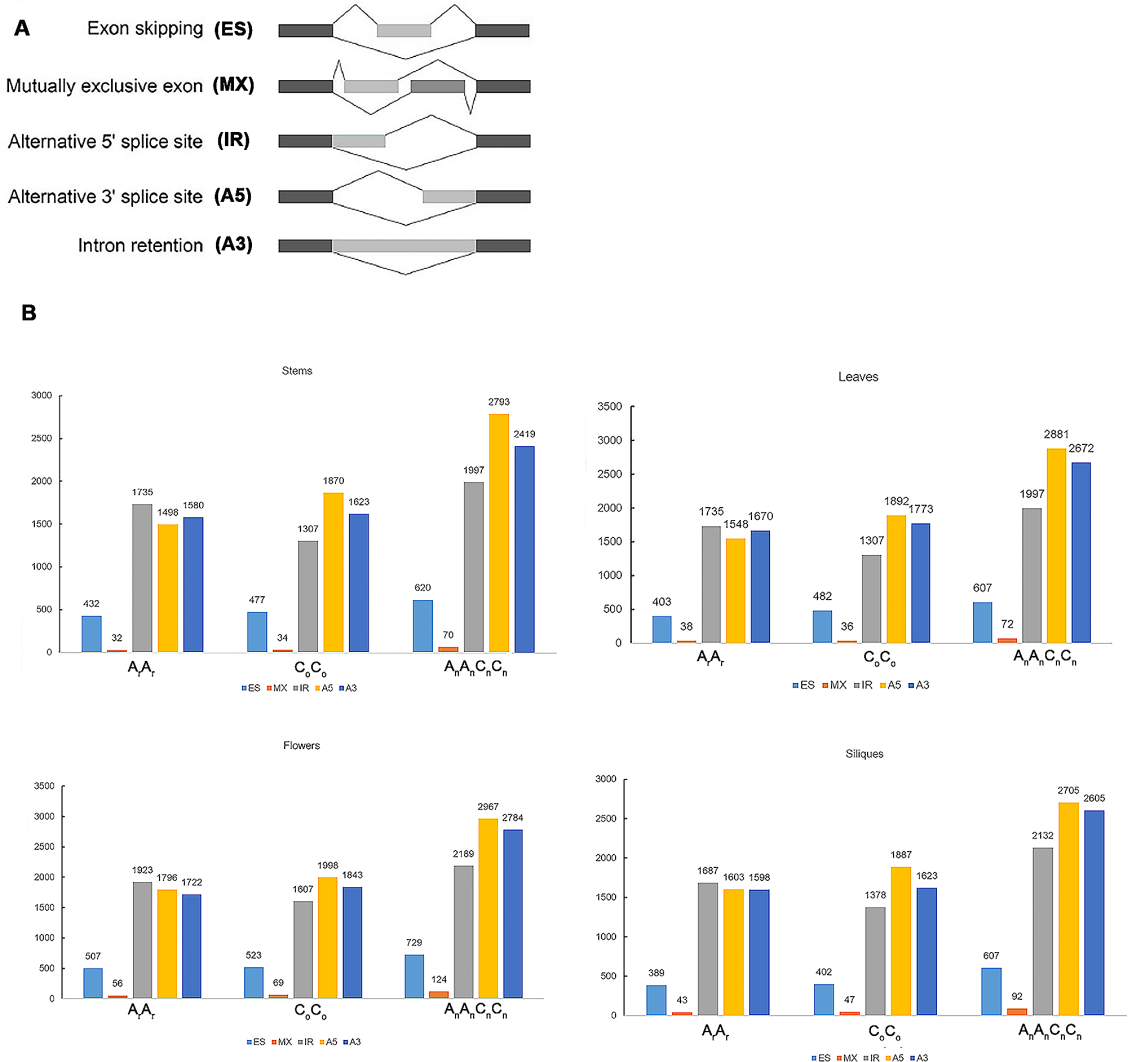
Characterization of alternative splicing (AS) events. (**A**) Visualization of five AS modes. (**B**) Numbers of different types of AS in four tissues from three species (*B. rapa*, *B. oleracea*, *B. napus*)

The number distribution of the five alternative splicing types of *B. napus* and its diploid progenitors in four tissue*s* can be obtained from Fig. 3. It can be seen from Fig. 3 that among the five types, IR, A5 and A3 have the largest number (about 5000–8000), followed by ES (400–600), and MX (30–120). The number of alternative splicing in flowers is the highest among the four tissues. These abundant alternative splicing produce multiple transcripts and thus regulate the flowering time of plants. The following observations can be made from Fig. 3: Three types of alternative splicing, IR, A5 and A3, are widely distributed in *B. napus* and its diploid progenitors. Among the three types, the number of naturally formed *B. napus* was the highest, followed by *B. oleacea* and *B. rapa*, suggesting that the number of alternative splicing increased during allopolyploidization and subsequent evolution, which may contribute to the enhancement of plant adaptability to the environment. During allopolyploidization, the number of alternative splicing events of IR type increased the most, suggesting the important role of this type of alternative splicing events in allopolyploidization.

### *Cis/trans* regulation of homoeologous gene expression in *B. napus* of four tissues

**Fig. 4:**
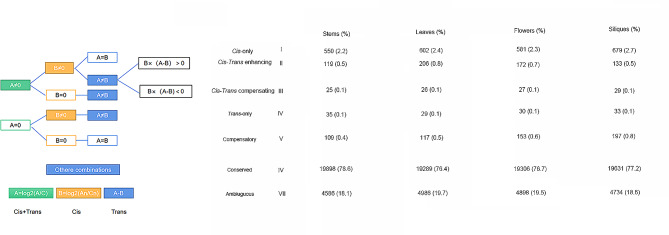
*Cis/trans* regulation of homoeologous gene expression in four tissues. Gene pairs were assigned to one of seven regulatory categories using the read counts of gene pairs in parents/progenitors (represented by A_r_-C_o_) and progeny (A_n_A_n_C_n_C_n_). A, the expression divergence in parents/progenitors; B, the expression divergence in progeny

A *cis/trans* regulation mechanism for gene expression was explored by dividing gene pairs into seven categories (Fig. 4). A_r_-C_o_/A_n_A_n_C_n_C_n_ had the highest proportion of gene pairs exhibiting conserved regulation (category VI), suggesting that gene expression was regulated conservatively during evolution. The number of gene pairs belonging to category VI in A_n_A_n_C_n_C_n_ was significantly lower than in A_r_-C_o_ (Chi squared test, *P* < 2.29 × 10^–16^), indicating that most gene pairs have evolved divergent regulation (categories I–V) after evolution rather than convergent regulation (category VI). The number of gene pairs belonging to category I (2.4%) accounted for more number of divergent regulation pairs, whereas the number for category II (0.6%) was much lower, suggesting that *cis* action tends to play a role independently while *trans* action does not. According to A_r_-C_o_, when *cis* and *trans* effects work together, the number of gene pairs showing opposing directions (category IV and V) was lower than the number of gene pairs showing the same direction (category III), and the opposite was observed in A_n_A_n_C_n_C_n_. Compared to A_r_-C_o_, A_n_A_n_C_n_C_n_ had a significantly higher number of gene pairs belonging to categories II-V (Chi-squared test, *P* < 0.05), with category III showing the greatest increase. It indicates that the conserved regulation of gene pairs (category VI) in A_r_-C_o_ was mainly converted to enhancing regulation (category III) when they converted to divergent regulation during evolution.

### Regulation of gene expression by methylation in *B. napus* and its diploid progenitors of four tissues

**Fig. 5:**
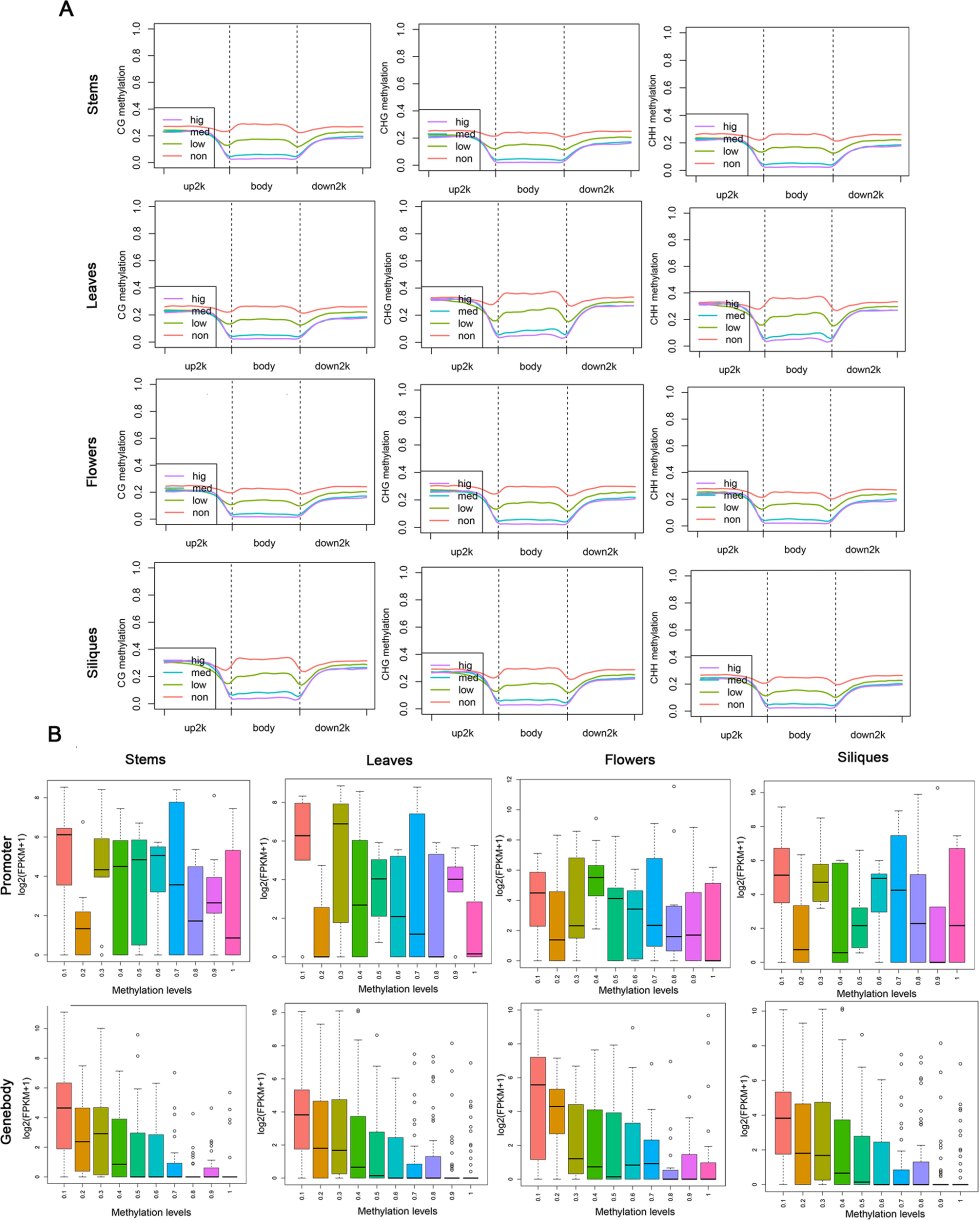
(**A**)Map of methylation levels of different gene expression levels in four tissues. Gene expression levels were divided into four levels: high, medium, low and non-high. Red, green, blue and purple were used to indicate the methylation levels of genes with different expression levels at different locations. (**B**) Different methylation levels correspond to the distribution of gene expression in four tissues

As an important epigenetic mode, DNA methylation plays an important role in gene expression. All samples were counted for methylation sites and their locations to study the relationship between methylation and gene expression (Fig. 5). It can be found that in promoter, there is no obvious correlation between methylation levels and gene expression levels among the four organs; genes with different methylation levels in stems and leaves are evenly distributed, and genes with methylation levels of 0.6–0.7 are the most distributed, while genes with different methylation levels in flowers and siliques are not evenly distributed. The methylation level was mainly concentrated at 0.5–0.8 in flowers and 0.8–0.9 in siliques. In gene body, methylation level is negatively correlated with gene expression level. The results indicated that the number of methylation sites in the gene body might affect the expression level. The higher the number of methylation sites in gene body, the lower the gene expression level. The number of methylation sites in the gene body upstream 2000 bp and downstream 2000 bp might influence gene expression, while the effect could be random, independent of the number of methylation sites. First of all, DNA methylation level was divided into high and low frequencies (non was the expression level < 0.001, low was the expression level between 0.001 and 5, medium was the expression level between 5 and 50, high was the expression level above 50), and then the changes of methylation level were counted. As can be seen from Fig. 5, The gene expression levels of the four tissues were consistent in promoter and gene body regions. Methylation levels were negatively correlated with gene expression levels, especially in the vicinity of TSS and TES (transcription start and end sites) (Fig. 5), and the methylation levels decreased with the increase of expression levels. The differentially methylated and differentially expressed genes were extracted for GO enrichment analysis. It can be known from Table S1, S2, S3 and S4, the enrichment is in three categories (biological process, molecular function and cellular component). In stems and leaves, the most enriched GO term is ATP binding (GO:0005524); in flowers, RNA binding (GO:0003723); and in siliques, heme binding (GO:0020037).

### Generational transmission analysis of expression and DNA methylation patterns

**Fig. 6:**
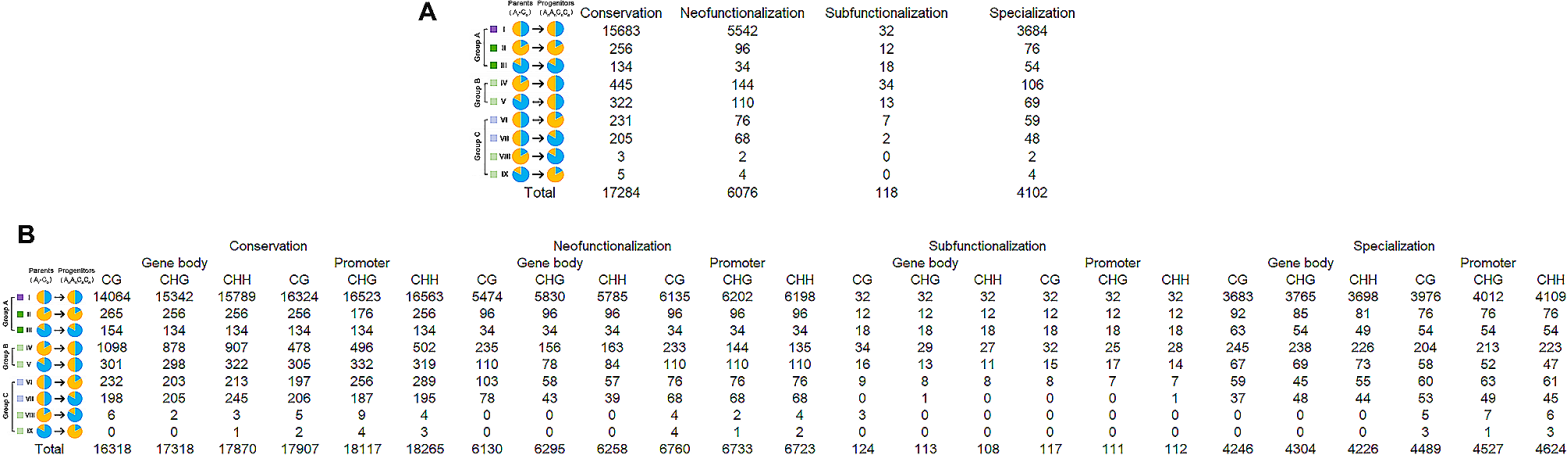
Generational transmission of expression and epigenetic modification patterns of homoeologous gene pairs. Numbers of homoeologous gene pairs exhibiting different expression (**A**) and DNA methylation patterns (**B**) in A_r_-C_o_/A_n_A_n_C_n_C_n_. Blue, A ortholog/homoeolog; yellow, C ortholog/homoeolog

As part of the study, we divided the gene pairs represented in four tissues, A_r_-C_o_/A_n_A_n_C_n_C_n_, into three groups, each with nine patterns of gene expression/epigenetic modification (Fig .6A). For Group A (patterns I-III), orthologous genes expressed in parents and progenitors were inherited (parental legacy); group B (pattern IV-V) consisted of gene pairs for which initial expression; group C (pattern VI-IX) consisted of gene pairs with biased expression which were novel. According to the same classification method we also have statistics generational transmission analysis of DNA methylation patterns (Fig. 6B). Group A represented the highest proportion of gene pairs, and patterns VIII and IX represented the lowest proportion (Fig. 6A). It was evident that parental legacy played a tremendous role in transmitting gene expression and DNA methylation patterns. However, it is worth mentioning that this phenomenon is not obvious in subfunctionalization genes. Further, we divided the nine patterns into two categories based on whether ortholog A and ortholog C differed in expression/DNA methylation patterns in parents/progenitors (Fig. 6A). In Fig. 6A, the state in parents/progenitors is indicated with a dark color if it is maintained in progenies; otherwise, it is indicated with a light color. According to our findings, homoeologous gene pairs in progenies tend to maintain this relative state (parental legacy) when there are no statistical differences in their expression/DNA methylation (97.03% and 97.29% homoeologous gene pairs ), homoeologous gene pairs in progenies tended to maintain this relative state (parental legacy). When there is a statistical difference in the expression/DNA methylation status of two homologous genes in the parent/ancestor, the homologous gene pairs in the offspring tend to become statistically non-different, followed by the maintenance of this relative state (parental legacy), and the cases of opposite differences are the least.

## Discussion

Although angiosperms have many duplicate genes, and they play a prominent role in evolution [44–48], their paths from genetic redundancy to functional divergence and long-term retention remain unclear. Many angiosperms are unique in that they are self-pollinating, which may reduce their adaptive potentials [49–52], as a result, duplicate genes cannot diverge evolutionaryly. Due to the lack of approaches for assessing functional divergence after duplication until recently, no genome-wide studies have been conducted to understand how duplicate genes evolve and are retained over long evolutionary periods. Additionally, previous studies in angiosperms have mainly focused on duplicates derived from WGD, whereas little attention has been paid to how evolution occurs after SSD. In angiosperms, our study represents the first genome-scale analysis of functional evolution following SSD. According to an analysis of expression profiles in four tissues of *B. rapa*, *B. oleracea*, and *B. napus*, SSD is primarily characterized by functional conservation over time. The preservation of duplicate genes may be the product of negative selection, which plays a role in preserving ancestral functions in both copies due to the benefit of increasing gene dosage [11]. If preservation is the result of non-allelic switching, it may be the result of slower functional differentiation due to reduced selection efficiency [13, 14]. In *B. napus* and its diploid progenitors, one or both of these mechanisms may inhibit evolutionary divergence of duplicate genes. Despite our study’s focus on SSD, analyses of WGD often suggest increased gene dosage as a mechanism for replicating genes. The results of our analysis suggest that most Brassica duplicates have been functionally conserved, but many WGD- and SSD- derived duplicates appear to have suffered functional divergence. Previous studies on *Arabidopsis thaliana* have shown that WGD and SSD- derived repeats have greater sequence and expression differences than WGD repeats of the same age [10], which may be attributed to relaxed constraints [10]. Therefore, it is not surprising that in the species considered here, WGD and SSD-derived repeats may have functionally deviated from their ancestral state, and similar studies of WGD-derived repeats may reveal similar trends observed in *A. thaliana*. In addition, we found that difference in the expression of WGD and SSD- derived *Brassica* duplicates are asymmetrically induced mainly by neofunctionalization, as has been uncovered in grass [53].

Through analysis of *cis-trans* regulation, it can be concluded that in the four tissues of *B. napus* and its diploid progenitors, the number of *cis*-only regulation is greater than that of *trans*-only regulation (~ 2.4% vs. ~ 0.6%), which is the same as in cotton [54]. However, the proportion of *cis* and *trans* effects on gene differences reported in maize and teosinte was (~ 45% vs. ~ 55%) [55], and these observations suggest that the two regulatory mechanisms are almost equally important in evolution, and that even in cotton, a smaller number of trans effects is more associated with expression differences. In our study, we found that DNA methylation in gene promoter region regulates gene expression, but coding DNA methylation has no significant effect on gene expression, which is similar to the report in wheat [56]. Although promoter DNA methylation generally inhibits gene transcription, it can also promote gene transcription in some cases, such as the ROS1 gene in *Arabidopsis* [57] and some genes that inhibit tomato fruit ripening [58]. In conclusion, the molecular mechanism of DNA methylation regulating the expression of genes related to plant growth and development is different. The homologous expression bias of allopolyploids has been extensively studied, but the effect of differentiation of gene fate on them is unknown. Here, we comprehensively analyzed the skewed gene expression and asymmetric DNA methylation modification of homologous pairs of A_r_-C_o_ and A_n_A_n_C_n_C_n_. One of our concerns is how homologous gene expression/DNA methylation modification is reshaped after allopolyploidization in *B. napus*. We found that parental inheritance is dominant in these remodeling processes (Fig. 6A), and a similar phenomenon has been found in other studies [59, 60]. Our results further support the idea that subgenome dominance in allopolyploids is primarily inherited from their progenitors, rather than being the outcome of allopolyploidization [60]. Through further analysis, it was found that although the differentiation of gene fate can lead to subgenome asymmetry. However, many homologous pairs that are asymmetrically expressed in parents also revert to no significant difference in offspring due to gene fate differentiation.

### Electronic supplementary material

Below is the link to the electronic supplementary material.

Supplementary Material 1

Supplementary Material 2

Supplementary Material 3

Supplementary Material 4

Supplementary Material 5

## Data Availability

All data related to this manuscript can be found within this paper and its Supplementary data. The raw data of RNA-seq reads were deposited in the NCBI database under accession number (SRR7816633-SRR7816668). The raw data of WGBS reads were deposited in the GSA database under accession number (CRA013177).
