# Supplementary material for: Characteristics of duplicated gene expression and DNA methylation regulation in different tissues of allopolyploid Brassica napus
Source: BMC Plant Biol. 2024 Jun 8;24:518. doi: 10.1186/s12870-024-05245-8 (PMC11162574; doi:10.1186/s12870-024-05245-8)
Supplement: Supplementary file 5 — Supplementary Material 5 [file 12870_2024_5245_MOESM5_ESM.docx]

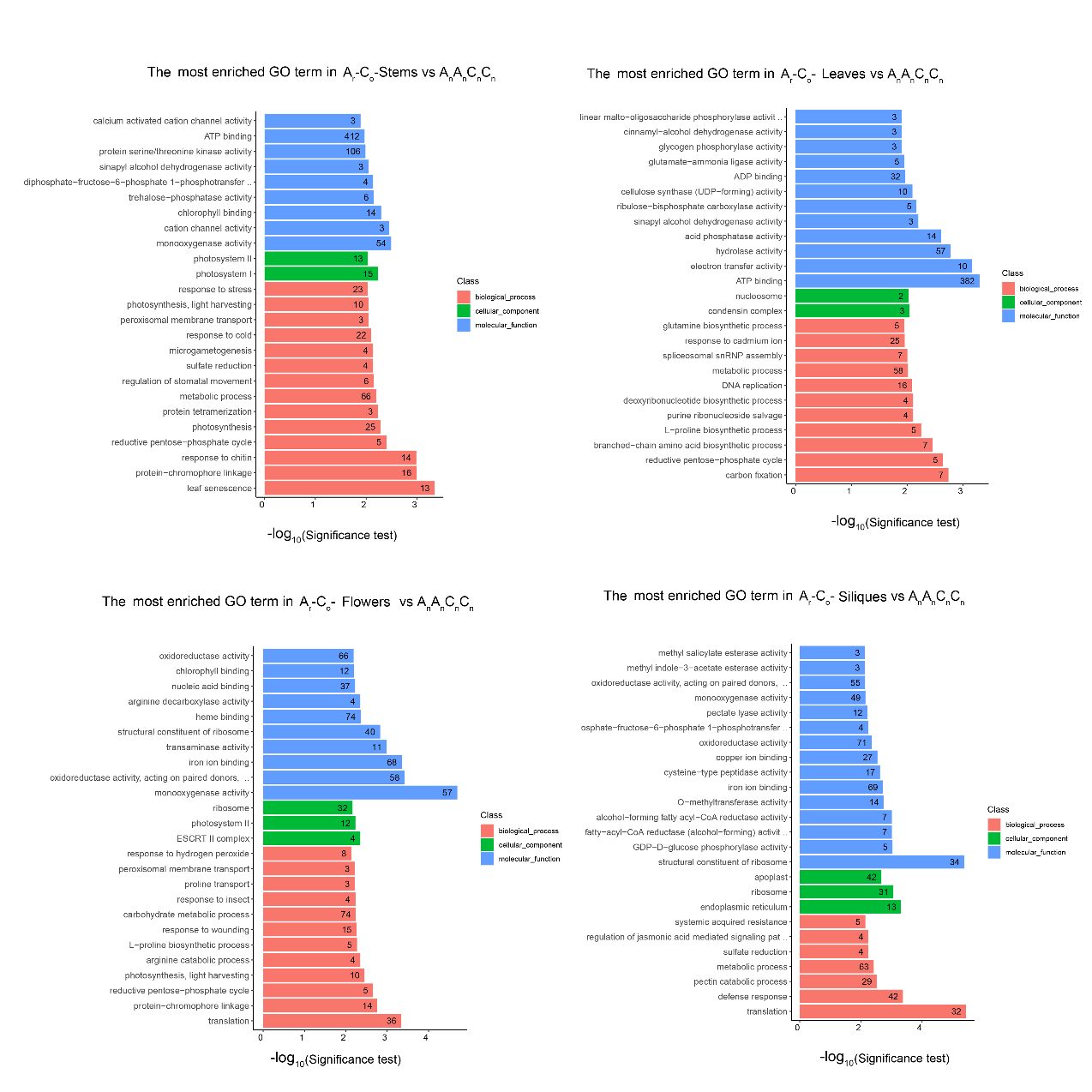


**Fig. S1** The most enriched GO term in four tissues (A_r_-C_o_ vs. A_n_A_n_C_n_C_n_). The differential genes of A_r_-C_o_ vs. A_n_A_n_C_n_C_n_ gene expression were analyzed, and then the differential genes of A_r_-C_o_ vs. A_n_A_n_C_n_C_n_ methylation were analyzed, and their intersection was found, and then these genes were used for GO term analysis. The top 25 most obvious GO terms are shown in the figure.


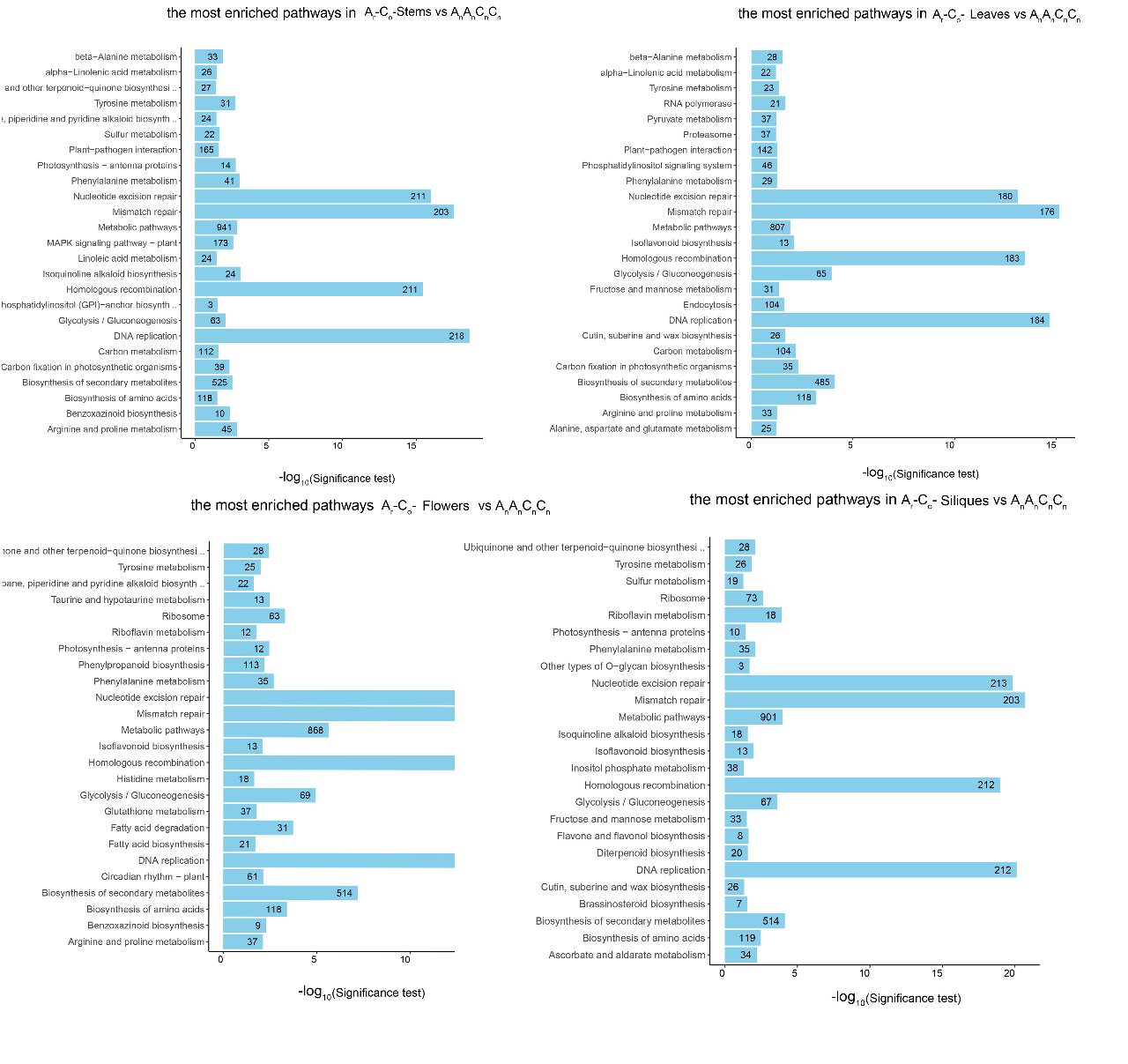


**Fig. S2** The most enriched KEGG Pathways in four tissues (A_r_-C_o_ vs. A_n_A_n_C_n_C_n_). The differential genes of A_r_-C_o_ vs. A_n_A_n_C_n_C_n_ gene expression were analyzed, and then the differential genes of A_r_-C_o_ vs. A_n_A_n_C_n_C_n_ methylation were analyzed, and their intersection was found, and then these genes were used for KEGG pathways analysis. The top 25 most obvious KEGG pathways are shown in the figure.
